# Burnout syndrome in primary and secondary school teachers in southern Brazil

**DOI:** 10.47626/1679-4435-2020-519

**Published:** 2021-02-11

**Authors:** Beatriz Maria dos Santos Santiago Ribeiro, Júlia Trevisan Martins, Rita de Cassia de Marchi Barcellos Dalri

**Affiliations:** 1 Departamento de Enfermagem, Universidade Estadual de Londrina - Londrina (PR), Brazil; 2 Escola de Enfermagem, Universidade de São Paulo - Ribeirão Preto (SP), Brazil

**Keywords:** teacher, professional exhaustion, work

## Abstract

**Introduction:**

Burnout syndrome is a form of professional exhaustion to which teachers are particularly vulnerable.

**Objectives:**

To investigate the association between burnout syndrome and occupational factors in primary and secondary school teachers in Brazil.

**Methods:**

A quantitative study of 200 teachers was conducted using a demographic and occupational questionnaire as well as the Maslach Burnout Inventory to investigate burnout syndrome. Data were analyzed using descriptive statistics and Mann-Whitney U tests.

**Results:**

Significant differences were observed in the burnout scores of teachers with different lengths of service in their current schools, as well as those with different lengths of teaching experience. Burnout syndrome scores also varied significantly between teachers with different types of work contracts and weekly workloads. No associations were observed between burnout scores and type of work shift, perceived professional recognition or teaching level.

**Conclusions:**

The present study identified significant associations between indicators of burnout syndrome and occupational factors such as length of employment in a given institution, length of teaching experience, type of work contract, hours worked and working at multiple institutions. These results underscore the vulnerability of teachers to burnout syndrome. Our findings also highlight the need to plan and implement initiatives to prevent burnout and maximize quality of life at work, with a special focus on the mental health of teachers.

## INTRODUCTION

The literal meaning of the word *burnout* is “to be burnt up or consumed.” The term also refers to feelings of exhaustion or depletion. In occupational settings, the word *burnout* is synonymous with work-related stress^1^; which comprises feelings of physical and mental exhaustion, irritability, loss of interest in work and decreased self-worth.^2^

In clinical psychology, the study of burnout syndrome (BS) involves the identification of factors which may be related to psychological distress in workers. This syndrome can also be viewed from a social perspective, since stressors can be a consequence of the work environment.^3^

BS is a complex condition whose impact is difficult to measure due to underreporting and symptom overlap with other disorders. False-positive diagnoses of depression and anxiety disorders, or false negative diagnosis of BS itself, can be problematic for patients, resulting in inadequate treatment or insufficient insurance coverage.^1^

Although all workers are susceptible to BS, this condition is especially prevalent in teachers due to their daily exposure to stressors such as high productivity demands, heavy workloads, crowded classrooms, the need to take work home, lack of social and leisure activities, absence of professional recognition, low salaries and poor working conditions, all of which may lead to overwork and illness. These factors also contribute to the increased likelihood of BS among teachers.^4^

Within school settings, a significant number of teachers move away from the classroom due to stress, depression, fatigue, physical and emotional exhaustion, and panic disorder. This is often prompted by situations faced by teachers in their occupational practice, such as direct contact with students’ emotional and academic issues, overcrowded classrooms, student indiscipline, low salaries, long hours, threats from students and their families, and bureaucratic interference in everyday activities.^5^

BS can interfere with the educational process and create problems at a national level, so that a study of this phenomenon in a medium-sized town may be especially relevant. Few studies have been conducted in cities of this size, although working conditions in smaller towns are known to differ from those observed in large cities.

In light of the aforementioned findings and the features of Brazilian society, it is especially pertinent to investigate BS in teachers in order to contribute to initiatives that promote a healthy work environment, contributing to the prevention and management of illness and injury in this population. The aim of this study was therefore to investigate the association between BS and the occupational characteristics of primary and secondary school teachers in Brazil.

## METHOD

A quantitative study of state school teachers was conducted between October 2018 and March 2019 in a medium-sized town in the state of Paraná, Brazil. The study population consisted of 393 teachers. The sample size required to detect a significant effect with a 95% confidence interval in a finite population of this size would be 192 teachers. The final sample for the present study consisted of 200 teachers.

Inclusion criteria consisted of current employment as a primary or secondary teacher at a state school, in any shift, and at least 6 months’ teaching experience. Exclusion criteria included being on leave or vacation during the study period, and working with other forms of teaching, such as special education or professional training outside of school settings.

Data were collected using Google Forms, with an electronic survey that remained available to participants for a period of 5 months. Data collection involved the following steps: 1) in-person and telephone recruitment; 2) provision of access to survey; 3) respondent count; 4) recruitment of additional participants; and 5) achievement of required sample size.

Participants completed a sociodemographic and occupational questionnaire, as well as the Maslach Burnout Inventory (MBI). This instrument contains 22 Likert-type items divided into three subscales: emotional exhaustion (nine items), depersonalization (five items) and professional accomplishment (eight items). The license to use the inventory was acquired from the Mind Garden company, which also authorized its use in the present study.

The MBI was used to evaluate the following dimensions of BS: emotional exhaustion (EE), depersonalization (D) and professional accomplishment (PA), all of which were reported as mean values.^3^ In the emotional exhaustion scale, scores ≥ 27 indicate high levels of burnout, while scores of 19 to 26 suggest medium levels and scores ≤ 18 indicate low levels of burnout. In the depersonalization scale, scores ≥ 10 correspond to high levels of burnout, while scores of 6 to 9 suggest moderate levels and scores ≤ 5 indicate low levels of burnout. Unlike the two previous subscales, where higher scores indicate greater burnout, the professional accomplishment score is inversely related to the level of burnout reported. In this subscale, scores ≥ 40 suggest low levels of burnout, while scores of 34 to 39 suggest moderate levels and scores ≤ 33, high levels of burnout.^^6^,7^ Data were analyzed using descriptive statistics, and MBI scores were compared between subsets of participants using the Mann-Whitney U test.

This study was approved by the Research Ethics Committee of the Universidade Estadual de Londrina (UEL) under project number CAAE 87890218.4.0000.5231 and all participants provided written informed consent before entering the study. Software licenses for the MBI-HSS were acquired from Mind Garden Inc. who owns the copyright to the instrument.

## RESULTS

The sample consisted of 200 teachers, 77.5% of whom were female while 22.5% were male; 10.5% were aged 18 to 30 years, 33.5% were 31 to 40 years old, 29.0% were 41 to 50, 24.5% were 51 to 60 and 2.5% were aged 60 years or older. In response to a question on ethnicity, 76.0% of participants identified as white, 2.5% as black, 15.0% as mixed race, 2.0% as mulatto and 4.5% as Asian. Questions about number of offspring revealed that 35.5% of respondents had no children, 18.5% had one child, 31.5% had two children, 13.0% had three and 1.5% had four or more children. While 60.5% of participants were married or in a relationship, 39.5% were single, separated, or widowed.

Participants were also asked about length of employment at their current institution. A total of 26.5% of participants had been in their current schools for 5 years or less, while 24.0% had been at their current institution for 6 to 10 years and 49.5%, for 11 years or longer. Most of the sample (74% of participants) had over 11 years’ teaching experience. While 11.5% of participants had indeterminate contracts, 17.5% had fixed-term contracts, 8.5% were private contractors and 62.5% were public servants; 59.0% worked full-time, 37.5% worked part-time and only 3.5% worked on a different schedule (Table 1).

**Table 1:** Dimensions of burnout syndrome and occupational variables (n = 200), Apucarana (PR), Brazil, 2019.

Occupational profile	n	%	EE (M)	D (M)	PA (M)	Burnout	p-value
Length of current employment							
≤ 5 years	53	26.5	23.8	7.8	39.2	70.6	>0.05
6 to 10 years	48	24	30	10	36	76	>0.05
≥ 11 years	99	49.5	34	13	32	80	0.000*
Length of teaching experience							
≤ 5 years	31	15.5	30.6	13.8	31.8	76	>0.05
6 to 10 years	21	10.5	33	11	37	81	0.000*
≥ 11 years	148	74	29	10	37	76	>0.05
Type of work contract							
Indeterminate-term	23	11.5	30	11	32	72	>0.05
Fixed-term	35	17.5	28	9	38	75	>0.05
Private contractor	17	8.5	28	14	29	70	>0.05
Public servant	125	62.5	30	10	37	77	0.000*
Working hours							
Full time (40 hours/week)	118	59.0	28	10	37	75	>0.05
Part time (less than 40 hours/week)	75	37.5	31	11	35	77	0.003*
Other	7	3.5	34	10	35	80	>0.05
Work at more than one institution							
Sim	88	44.0	32	11	36	79	0.008*
No	112	56.0	28	10	36	74	>0.05
Shifts worked							
Morning	89	44.5	30	10	37	77	>0.05
Afternoon	62	31.0	30	11	37	77	>0.05
Evening	49	24.5	31	10	37	78	0.043*
Satisfaction with professional recognition							
Sim	125	62.5	27	10	36	74	>0.05
No	75	37.5	33	10	36	79	0.014*
Level taught							
Primary	35	17.5	26	9	36	72	>0.05
Secondary	28	14.0	29	9	34	72	>0.05
Primary and secondary	137	68.5	31	11	36	78	>0.05

D: depersonalization; EE: emotional exhaustion; M: mean; PA: professional accomplishment. Mann-Whitney U Test (p-value < 0.05).

* Significant values.

Additionally, 44.0% of participants worked in more than one institution, while 56.0% worked in a single school. While 44.5% of participants worked morning shifts, 31.0% worked in the afternoon and 24.5% worked at night. A question regarding professional recognition revealed that 62.5% of participants felt appropriately recognized for their work while 37.5% did not; 17.5% of respondents were primary school teachers, 14.0% were high school teachers and 68.5% of participants taught at both levels.

The results showed significant differences in burnout scores between teachers with over 11 years of employment at their current institution and the remainder of the sample. Participants with 6 to 10 years’ teaching experience also scored higher than their peers across the different dimensions of BS. Additionally, teachers employed as public servants or part-time workers (less than 40 hours a week) differed from other categories with regards to burnout scores (Table 1).

Interestingly, teachers working less than 40 hours a week showed higher levels of burnout than those working full-time (40 hours a week) or on other schedules. Working at more than one institution was also associated with a higher likelihood of burnout (Table 1). No significant differences were observed between the scores of teachers working different shifts, teachers with positive or negative perceptions of professional recognition and individuals teaching at different levels (Table 1).

## DISCUSSION

The sociodemographic characteristics of the present sample were similar to those of participants in a previous study, conducted in Simões, in the state of Piauí. In the latter study, most participants were also female (77.6%) and had a partner at the time of the study (59.2%); 6.1% of participants were 20 to 29 years old, 34.7% were 30 to 39, 36.8% were 40 to 49 years old and 22.4% were 49 years or older.^8^

The present study found that individuals who had been working in the same institution for over 11 years showed an increased likelihood of burnout*.* A similar pattern was observed for those with 6 to 10 years’ teaching experience. These findings differ from those obtained in a study conducted in Greece with 964 male and 2473 female primary and secondary school teachers; the study identified a correlation between length of teaching experience and resistance to occupational stress and symptoms of BS.^9^

The present study also noted that burnout levels were significantly higher among teachers working as public servants. These results differ from those of a previous study conducted in the state of Paraná, where teachers with fixed-term contracts reported higher workloads, longer hours and jobs at multiple schools, in addition to less decision-making autonomy than teachers employed as public servants, who could choose the schools in which they worked.^10^ Teachers with temporary contracts had poorer working conditions than those with permanent positions and may have been disappointed by the nature of their work environment, which could have led to dissatisfaction and suffering.^11^

Teachers who worked for less than 40 hours a week in their main job reported significantly higher levels of burnout. This is not a universal finding, as many studies report that professional exhaustion tends to be related to longer working hours.^12,13^

Working at multiple institutions was also associated with a greater likelihood of burnout. Excessive workloads often lead to emotional, creative, and physical exhaustion, which in turn has a negative impact on the energy levels, efficiency, health, and well-being of workers.^12^ In line with the present findings, Demeneck and Kurowski^13^ note that excess work can lead to BS as a result of chronic stress and intense mental exhaustion due to professional overload.

Although in the present study, BS was not significantly associated with weekly working hours, it was associated with having more than one job. Burnout scores were not associated with the particular shift worked by teachers. However, a recent literature review found that BS is often associated with shift or night work.^13^ While professional recognition was also unrelated to burnout scores in the present study, it is considered a protective factor against this condition, since the absence of recognition can lead to sadness, apathy, discouragement and emotional exhaustion.^14^

The level of teaching was also unrelated to burnout scores. However, teachers at all levels can be susceptible to BS.^3^ Both primary and secondary teachers have a high risk of burnout, since they experience constant human interaction and, consequently, an increased likelihood of exposure to interpersonal conflict and violence, in addition to a lack of recognition.^15,16^

Some teachers, however, show resilience in the face of factors which can lead to BS. Resilience is a combination of cognitive and behavioral strategies that aim to control, minimize, or accept stressful and conflicting situations.^1^ These strategies may include individual temperament or personality traits, as well as problem-solving skills.^17^ Resilience is associated with self-regulating mechanisms that differ from person to person, depending on an individual’s overall health, personality, intelligence, goal-setting ability, self-efficacy beliefs, emotional regulation and stress management strategies.^18^

The present study may have been limited by its cross-sectional design, which prevents the identification of causal relationships between variables. This topic should be further investigated in studies with larger samples, involving teachers from other cities and regions in Brazil, as well as a research design that could reveal causal relationships between variables.

## CONCLUSIONS

The present study identified significant associations between indicators of BS and several occupational factors, such as length of employment in a given institution, length of teaching experience, type of work contract, hours worked and working at multiple institutions. These findings highlight the need to plan and implement initiatives to prevent burnout and maximize quality of life at work, with a focus on mental health.

This study contributes to public policy debates about initiatives to improve teacher health and may enable teachers and school administrators to reflect on the importance of making changes to the organizational environment.

## References

[1] Medanha MH, Bernardes PF, Shiozawa P (2018). Desvendando o burn-out: uma análise interdisciplinar da síndrome do esgotamento profissional.

[2] Tavares KFA, Souza NVDDO, Silva LDD, Kestenberg CCF (2014). Ocorrência da síndrome de Burnout em enfermeiros residentes. Acta Paul Enferm. (Impr.).

[r3] Maslach C, Leiter MP (2016). Burnout. Stress: Concepts, Cognition, Emotion, and Behavior.

[4] Koga GKC, Melanda FN, Santos HD, Sant'Anna FL.González AD.Mesas AE (2015). Fatores associados a piores níveis na escala de Burnout em professores da educação básica. Cad Saúde Colet. (Online).

[5] Pomiecinski JAS, Pomiecinski CM (2014). Gestão escolar: uma reflexão sobre a saúde emocional do professor-entre o stress e a síndrome de burnout. Colóq. Int. Educ.

[6] Sa LOD (2002). Burnout e controlo sobre o trabalho em enfermagem oncológica: estudo correlacional.

[7] Santos JWD (2009). A síndrome de Burnout: uma análise social e psicodinâmica. Rev. Cient. Eletr. Psicol.

[8] Carvalho MDG, Oliveira GFD, Carita A (2016). Representações da Violência por Professores. ID on line Rev Psicol.

[9] Kourmousi N, Alexopoulos EC (2016). Stress sources and manifestations in a nationwide sample of pre-primary, primary, and secondary educators in Greece. Front public health.

[10] Ferreira DCK, Abreu CBDM (2014). Professores temporários: flexibilização das contratações e condições de trabalho docente/Temporary Teachers: flexibilization of contracts and teacher's work conditions. Trab. Educ. (Online).

[11] Both J, Nascimento JVD, Sonoo CN, Lemos CAF, Borgatto AF (2013). Bem estar do trabalhador docente em Educação Física ao longo da carreira. Rev. Educ Fis./UEM.

[12] Lacerda RB, Ferreira MBG, Bracarense CF, Sene LVD, Simões ALDA (2016). Contexto de trabalho e Síndrome de Burnout na equipe de enfermagem da Estratégia de saúde da família. Cult. cuid.

[13] Demeneck V, Kurowski C (2010). Síndrome de Burnout: ameaça da saúde mental do trabalhador. Cad PAIC.

[14] Moreira DDS, Magnago RF, Sakae TM, Magajewski FRL (2009). Prevalência da síndrome de burnout em trabalhadores de enfermagem de um hospital de grande porte da Região Sul do Brasil. Cad Saúde Públ.

[15] Carlotto MS (2002). A síndrome de burnout e o trabalho docente. Psicol Estud.

[16] Santos AAD, Nascimento Sobrinho CL (2012). Revisão sistemática da prevalência da Síndrome de Burnout em professores do ensino fundamental e médio. Rev. baiana saúde pública.

[17] Lee JH, Kim HY (2018). Symptom Distress and Coping in Young Korean Breast Cancer Survivors: The Mediating Effects of Social Support and Resilience. J Korean Acad Nurs.

[18] Baratta MV, Maier SF (2017). New tools for understanding coping and resilience. Neurosci Lett.

